# Supplementary material for: RPA shields inherited DNA lesions for post-mitotic DNA synthesis
Source: Nat Commun. 2021 Jun 22;12:3827. doi: 10.1038/s41467-021-23806-5 (PMC8219667; doi:10.1038/s41467-021-23806-5)
Supplement: Supplementary file 1 — Supplementary Information [file 41467_2021_23806_MOESM1_ESM.pdf]

Supplementary Information

RPA shields inherited DNA lesions for post-mitotic DNA synthesis

Lezaja et al.

Supplementary Figures 1 – 10

Supplementary Tables 1 – 2

Supplementary Figure 1

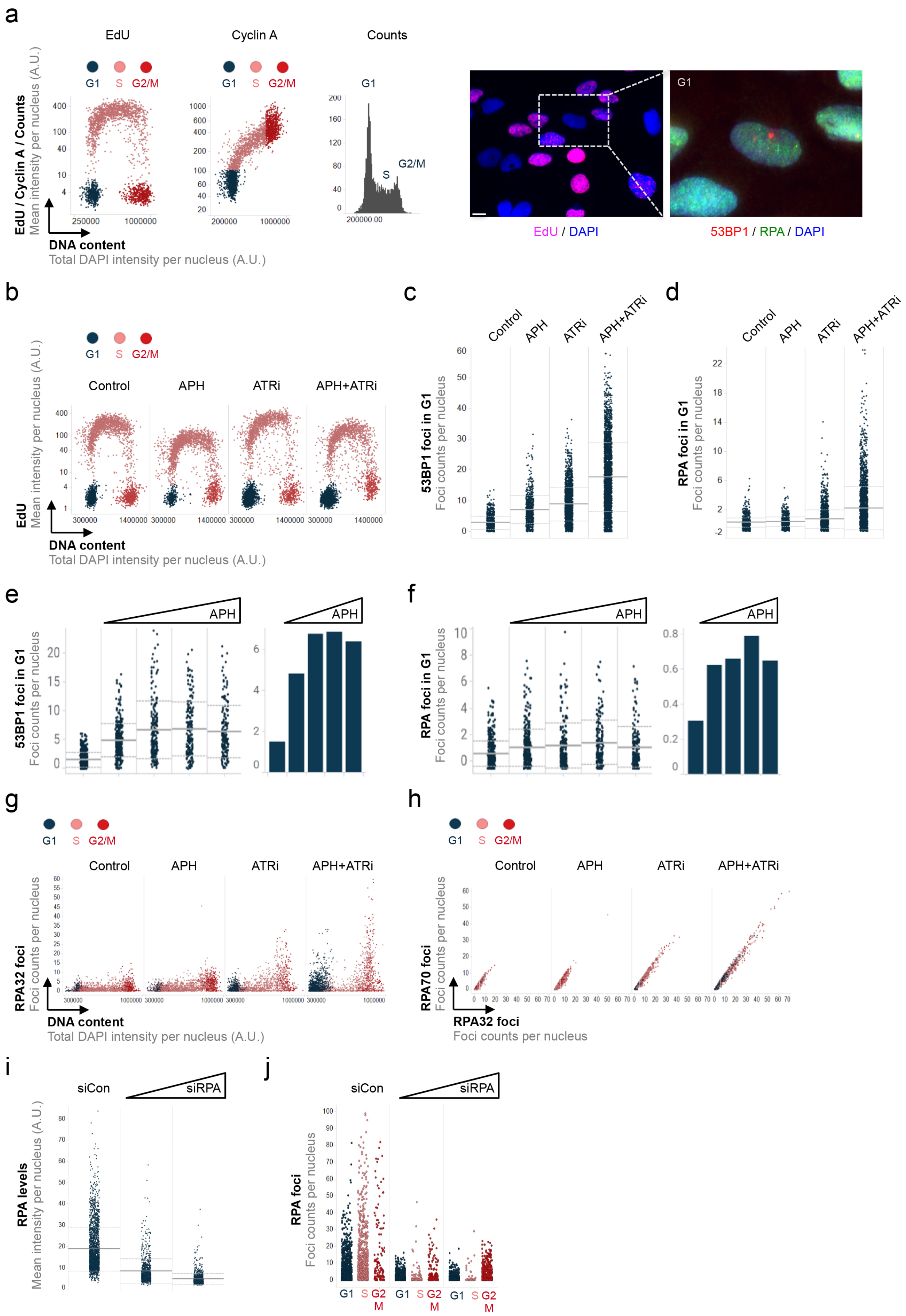

Supplementary Figure 1: Replication stress-associated RPA-marked lesions in G1. (a) Examples of microscopy-based cell cycle staging by QIBC, as used in this study. Briefly, asynchronously growing cell populations were fixed and microscopy images of at least 1000 cells per condition were acquired by high-content microscopy. The total DAPI intensity was used to measure DNA content (from 2N to 4N), and mean intensities of EdU and/or Cyclin A were used as additional parameters to define G1, S and G2/M. Images to the right provide examples, with a cell in G1 (EdU-negative, low DNA content) containing 53BP1 and RPA foci highlighted. **(b)** QIBC EdU profiles for cell populations as in Fig. 1a and b. The colour code is based on the EdU/DAPI profile. **(c)** Quantification of 53BP1 foci in at least 500 cells in G1 per condition, corresponding to Fig. 1a. Horizontal lines indicate means ± SD of single cell data. **(d)** Quantification of RPA70 foci in at least 500 cells in G1 per condition, corresponding to Fig. 1b. Horizontal lines indicate means ± SD of single cell data. **(e)** Quantification of 53BP1 foci in G1 after exposure to increasing APH concentrations (0, 0.2µM, 0.3µM, 0.4µM, 0.6µM). At least 150 G1 cells per condition were analysed. Horizontal lines indicate means ± SD of single cell data. **(f)** Quantification of RPA foci in G1 corresponding to (e). Horizontal lines indicate means ± SD of single cell data. **(g)** Quantification of RPA32 foci in a cell cycle resolved manner by QIBC based on DAPI and EdU. At least 1000 cells per condition were analysed. **(h)** Quantification of RPA32 foci and RPA70 foci in the same cell population in a cell cycle resolved manner by QIBC. G1 cells are labelled in blue, S phase cells in light red, and G2/M cells in dark red. RPA32 and RPA70 foci counts are compared. At least 1000 cells per condition were analysed. **(i)** U-2 OS cells were transfected with siRNA against RPA (0.2nM, 1nM) for 48h and treated with APH and ATRi for the last 24h. Cells were stained for RPA, EdU and DAPI and at least 500 cells per condition were analysed by QIBC. Mean RPA levels are depicted as control of the knockdown efficiency. Horizontal lines indicate means ± SD of single cell data. **(j)** Cell cycle resolved RPA foci analysis of the cells shown in (g). Scale bar: 10µm. A. U., arbitrary units. Source data are provided as a Source Data file.

Supplementary Figure 2

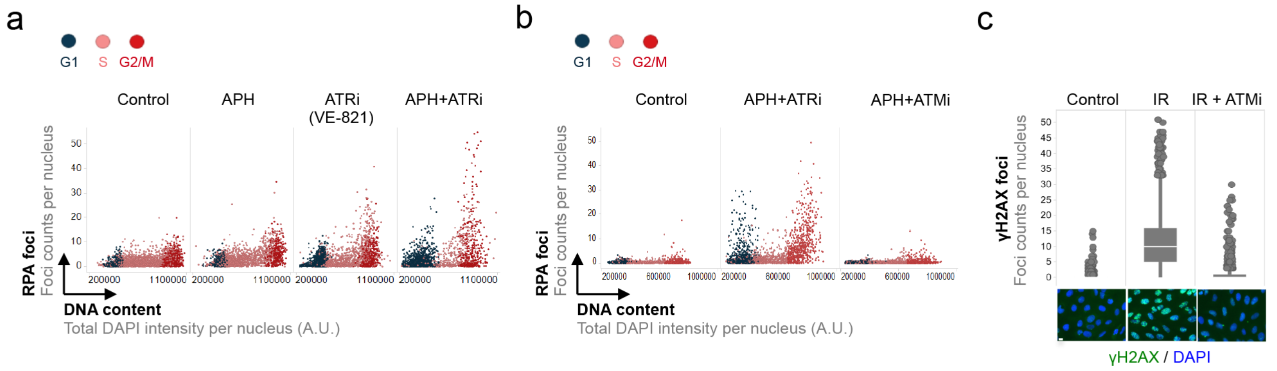

Supplementary Figure 2: ATR counteracts replication stress-associated RPA-marked lesions. (a) Asynchronously growing U-2 OS cells were treated as indicated, using a second ATR inhibitor (VE-821), and RPA foci formation was analysed in a cell cycle resolved manner by QIBC. At least 1000 cells per condition were analysed. **(b)** Asynchronously growing U-2 OS cells were exposed to APH combined with ATRi or ATMi for the last 8h. RPA foci formation was analysed in a cell cycle resolved manner by QIBC in at least 1000 cells per condition. **(c)** As a control for the ATMi, U-2 OS cells were treated with 0.5 Gy of IR in absence or presence of ATMi and fixed after 1h of recovery. Foci of γH2AX were analyzed by QIBC with n=2399 (Control), n=2446 (IR), n=2376 (IR+ATMi) cells per condition. Box plot with medians, boxes indicate the 25th and 75th centiles, whiskers indicate 5 and 95 per cent values. Representative images are shown below. Scale bar: 10μm. A. U., arbitrary units. Source data are provided as a Source Data file.

Supplementary Figure 3

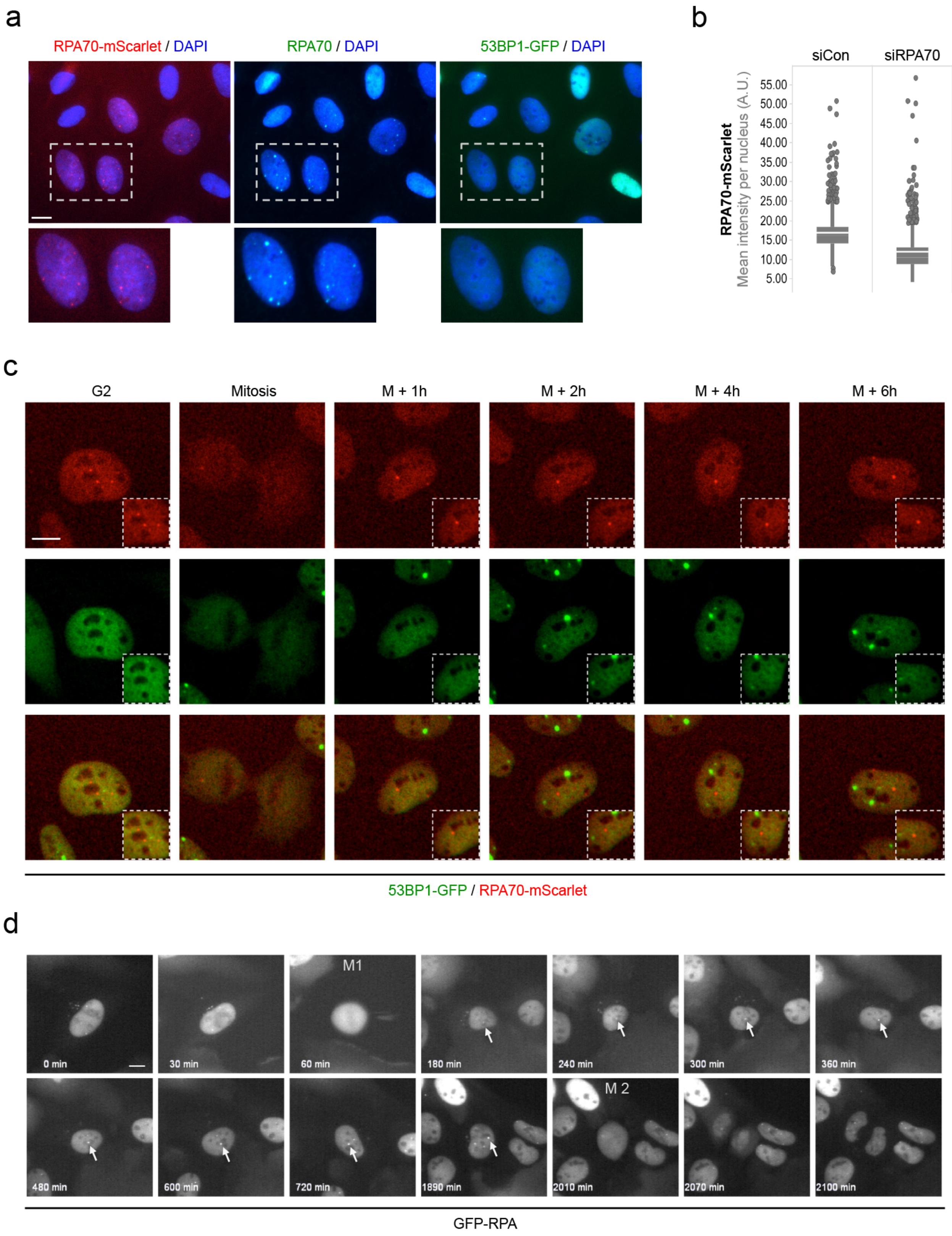

Supplementary Figure 3: 53BP1 and RPA mark distinct heritable DNA lesions. (a) Validation of U-2 OS 53BP1-GFP / RPA70-mScarlet cells, in which the endogenous *RPA70* gene locus had been engineered by CRISPR/Cas9 to express RPA70-mScarlet. Cells were fixed and stained for RPA70. Representative images of RPA70-mScarlet, antibody-based detection of RPA70, and GFP-53BP1 are shown. **(b)** U-2 OS 53BP1-GFP / RPA70-mScarlet cells were transfected with siRNA against RPA70 for 48h and mean RPA70-mScarlet intensities were measured by QIBC with n=1401 (siControl) and n=698 (siRPA70) cells per condition. Box plot with means is shown, boxes indicate the 25th and 75th centiles, whiskers indicate 5 and 95 per cent values. **(c)** Untreated asynchronously growing U-2 OS 53BP1-GFP / RPA70-mScarlet cells were followed through mitosis and into G1 by time-lapse microscopy at 30 minutes intervals. Note that RPA and 53BP1 form distinct foci in G1. **(d)** Untreated asynchronously growing U-2 OS RPA-GFP cells were followed through the cell cycle by time-lapse microscopy at 30 minutes intervals. The arrowhead points to an RPA focus, M1 indicates the first mitosis, M2 indicates the second mitosis. Scale bars: 10µm. A. U., arbitrary units. Source data are provided as a Source Data file.

Supplementary Figure 4

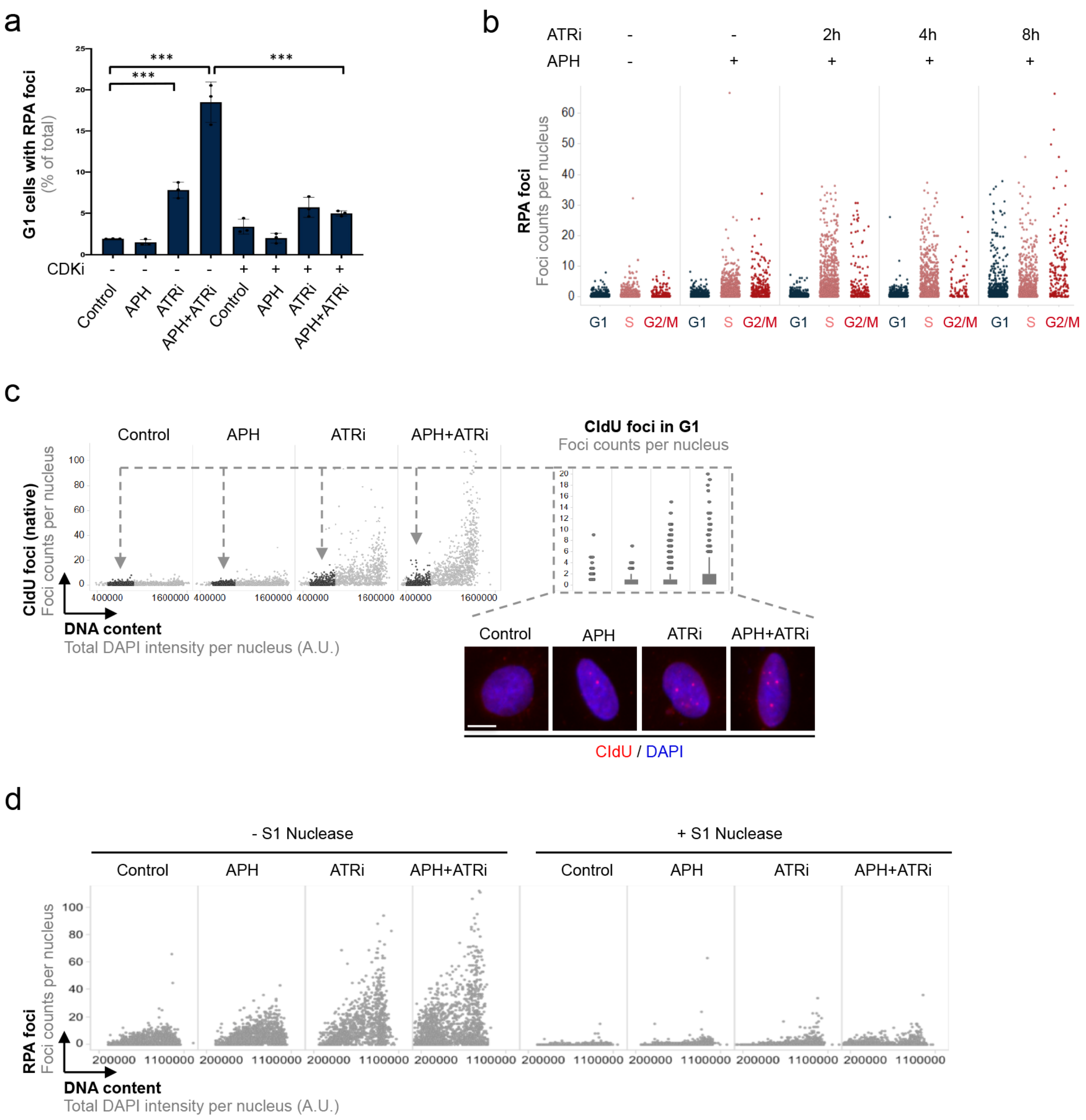

Supplementary Figure 4: RPA-marked lesions in G1 are associated with replication stress in the previous cell cycle. (a) U-2 OS cells were treated for 24h as indicated in absence or presence of CDKi RO-3306 to block mitotic cell division. The percentage of G1 cells positive for RPA foci was quantified in different treatment conditions from n=3 independent samples with n_1=4258, n_2=4161, n_3=4271 (Control), n_1=2936, n_2=3963, n_3=3925 (APH), n_1=4241, n_2=4882, n_3=4080 (ATRi), n_1=3390, n_2=3568, n_3=4080 (APH+ATRi), n_1=1661, n_2=1669 n_3=1258 (Control+CDKi), n_1=1866, n_2=1928, n_3=1457 (APH+CDKi), n_1=1452, n_2=1545, n_3=1651 (ATRi+CDKi), n_1=1724, n_2=1571, n_3=1954 (APH+ATRi+CDKi) cells per sample. P-values were determined by two-tailed unpaired t-test; *** p<0.001 (the exact p-values are p=0.0005, p=0.0003, p=0.0007, respectively). **(b)** U-2 OS cells were treated with APH for 24h as indicated in absence or presence of ATRi for the last 2h, 4h and 8h. Cells were stained for RPA, Cyclin A and DAPI and cell cycle resolved RPA foci formation was analysed by QIBC in at least 1000 cells per condition. **(c)** The thymidine analogue 5-Chloro-2′-deoxyuridine (CldU) was applied to cells for 24h prior to replication stress treatments. CldU was detected by QIBC in native (non-denaturing) conditions to measure ssDNA in a cell cycle resolved manner. CldU foci counts and representative images of cells in G1 are shown. At least 1000 cells per condition were analysed. **(d)** U-2 OS cells were treated for 24h as indicated, permeabilized and treated or not with S1 nuclease. RPA foci formation was analysed in a cell cycle resolved manner by QIBC in at least 1000 cells per condition. Scale bar: 10µm. A. U., arbitrary units. Source data are provided as a Source Data file.

Supplementary Figure 5

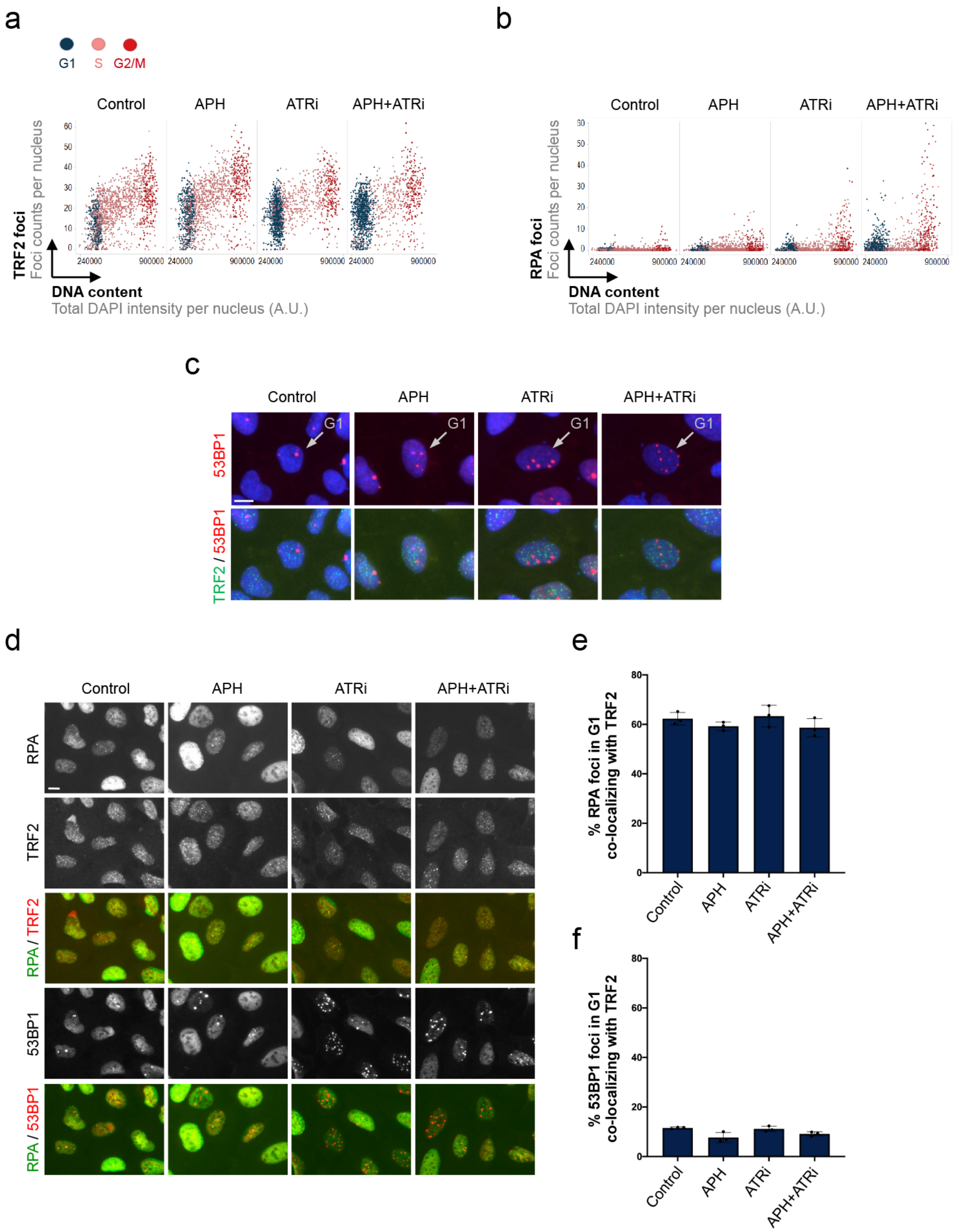

Supplementary Figure 5: RPA-marked inherited genomic lesions occur at telomeres. (a, b) QIBC-derived cell cycle resolved profiles of TRF2 foci (a) and RPA foci (b) in U-2 OS cells treated as indicated. At least 1000 cells per condition were analysed. **(c)** Representative images of individual G1 cells, treated as indicated and identified by QIBC-guided cell cycle staging, stained for 53BP1 and TRF2. **(d)** 4i experiment to sequentially stain RPA, TRF2, and 53BP1 in U-2 OS cells. Representative images of the sequential staining are shown. **(e)** Quantification of co-localization between RPA and TRF2 in G1 cells in different treatment conditions from n=3 independent samples with n_1=556 n_2=575, n_3=510 (Control), n_1=364, n_2=398, n_3=312 (APH), n_1=772, n_2=816, n_3=692 (ATRi), n_1=677, n_2=642, n_3=686 (APH+ATRi) cells in G1 per sample. Individual values and means ± SD are shown. **(f)** Quantification of co-localization between 53BP1 and TRF2 in G1 cells in different treatment conditions from n=3 independent samples with n_1=329 n_2=353, n_3=373 (Control), n_1=208, n_2=197, n_3=188 (APH), n_1=462, n_2=455, n_3=452 (ATRi), n_1=461, n_2=414, n_3=445 (APH+ATRi) cells in G1 per sample. Individual values and means ± SD are shown. Scale bars: 10µm. A. U., arbitrary units. Source data are provided as a Source Data file.

Supplementary Figure 6

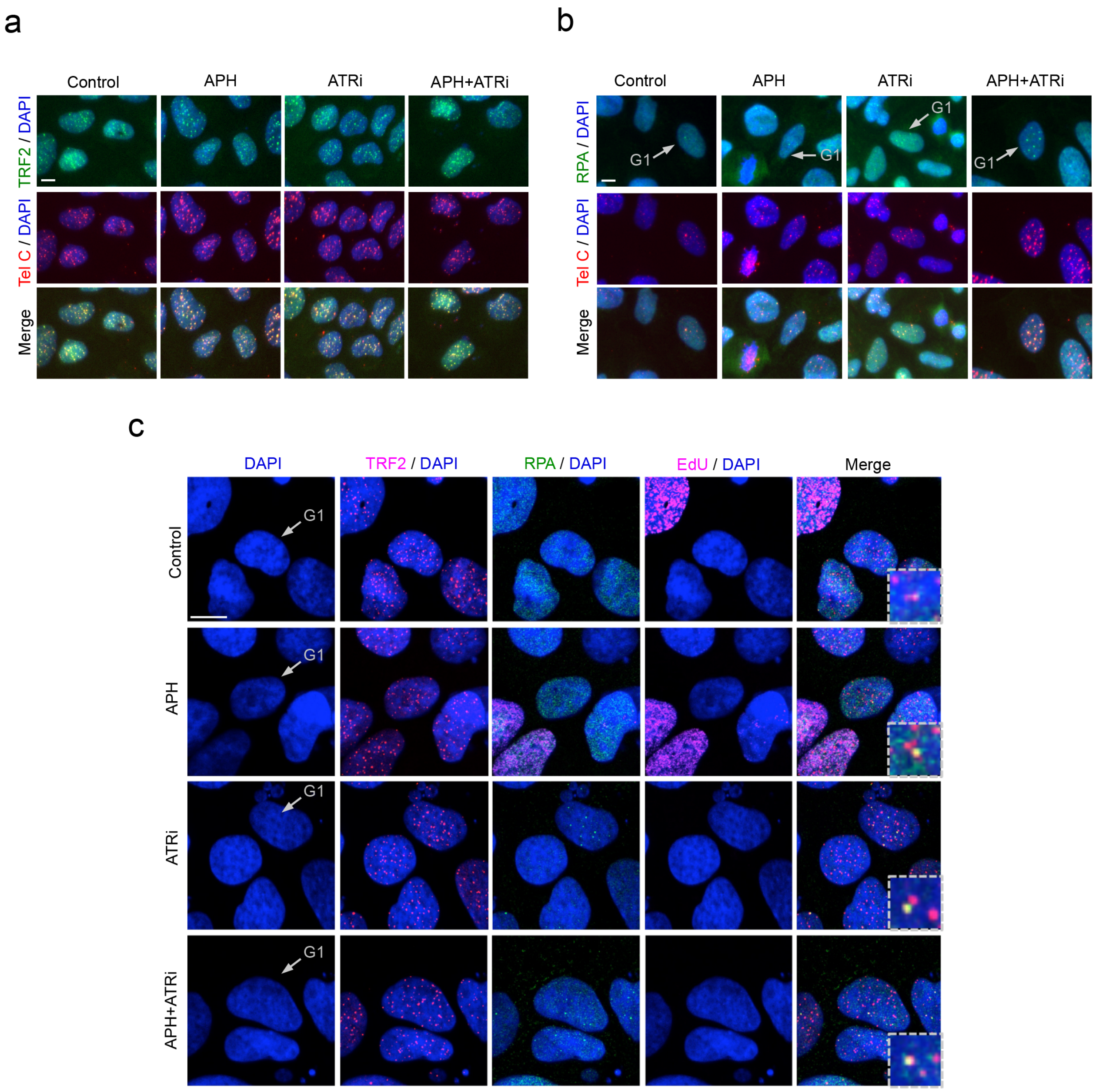

Supplementary Figure 6: RPA-marked inherited genomic lesions occur at telomeres. (a) Representative images of denaturing FISH-IF staining of U-2 OS cell after the indicated treatments, using a telomere-specific FISH probe (Tel C) and the TRF2 antibody to validate the specificity of the TRF2 signal. **(b)** Representative images of denaturing FISH-IF staining of U-2 OS cell after the indicated treatments, using the telomere-specific Tel C probe and the RPA antibody to validate the association of RPA foci in G1 cells with telomeres. **(c)** Single plane confocal images of U-2 OS cell after the indicated treatments showing RPA enrichment at telomeres in EdU-negative G1 cells. Scale bars: 10μm.

Supplementary Figure 7

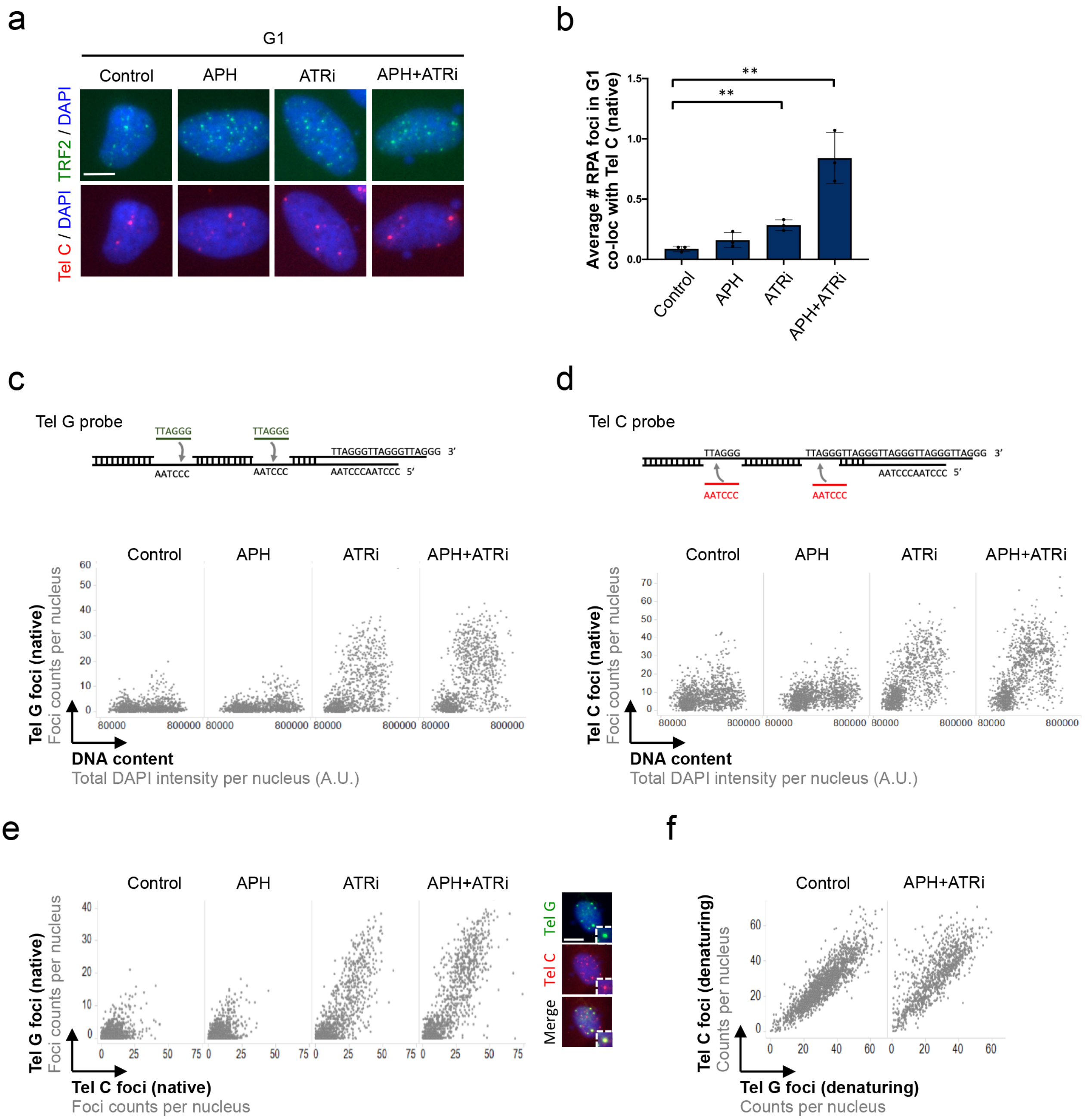

Supplementary Figure 7: Telomeres acquire heritable ssDNA lesions on both strands. (a) Representative images of native (non-denaturing) telomere FISH-IF in U-2 OS cells upon the indicated treatments using the telomere-specific Tel C probe and the TRF2 antibody. Note that Tel C foci under non-denaturing conditions increase in G1 cells upon replication stress and co-localize with TRF2 foci. **(b)** Quantification of the average number of RPA foci co-localizing with Tel C foci in G1 in different treatment conditions from n=3 independent samples with n_1=496, n_2=498, n_3=537 (Control), n_1=233, n_2=276, n_3=355 (APH), n_1=906, n_2=897, n_3=959 (ATRi), n_1=1038, n_2=1004, n_3=1131 (APH+ATRi) cells in G1 per sample. Individual average values and means ± SD are shown. P-values were determined by two-tailed unpaired t-test. ** p<0.01 (exact p-values are p=0.0021 and p= 0.0038, respectively). **(c)** Asynchronously growing U-2 OS cells were treated as indicated and native (non-denaturing) FISH was performed using telomere-specific Tel C and Tel G probes, which bind to single-stranded G-rich and C-rich telomere sequences, respectively. Scheme of the Tel G probe used for native telomere FISH. QIBC-derived cell cycle resolved scatter plots of Tel G foci after the indicated treatments. At least 1000 cells per condition were analysed. **(d)** As in (c) for the Tel C probe. **(e)** Correlation between the Tel G and Tel C FISH signals under native (non-denaturing) conditions from (c) and (d). Representative images for the APH+ATRi condition are shown to the right. **(f)** Correlation between the Tel G and Tel C FISH signals under denaturing conditions. Note that replication stress treatments only induce telomere FISH signals coming from single-stranded telomere sequences when FISH is performed under native (non-denaturing) conditions. Scale bars: 10µm. A. U., arbitrary units. Source data are provided as a Source Data file.

Supplementary Figure 8

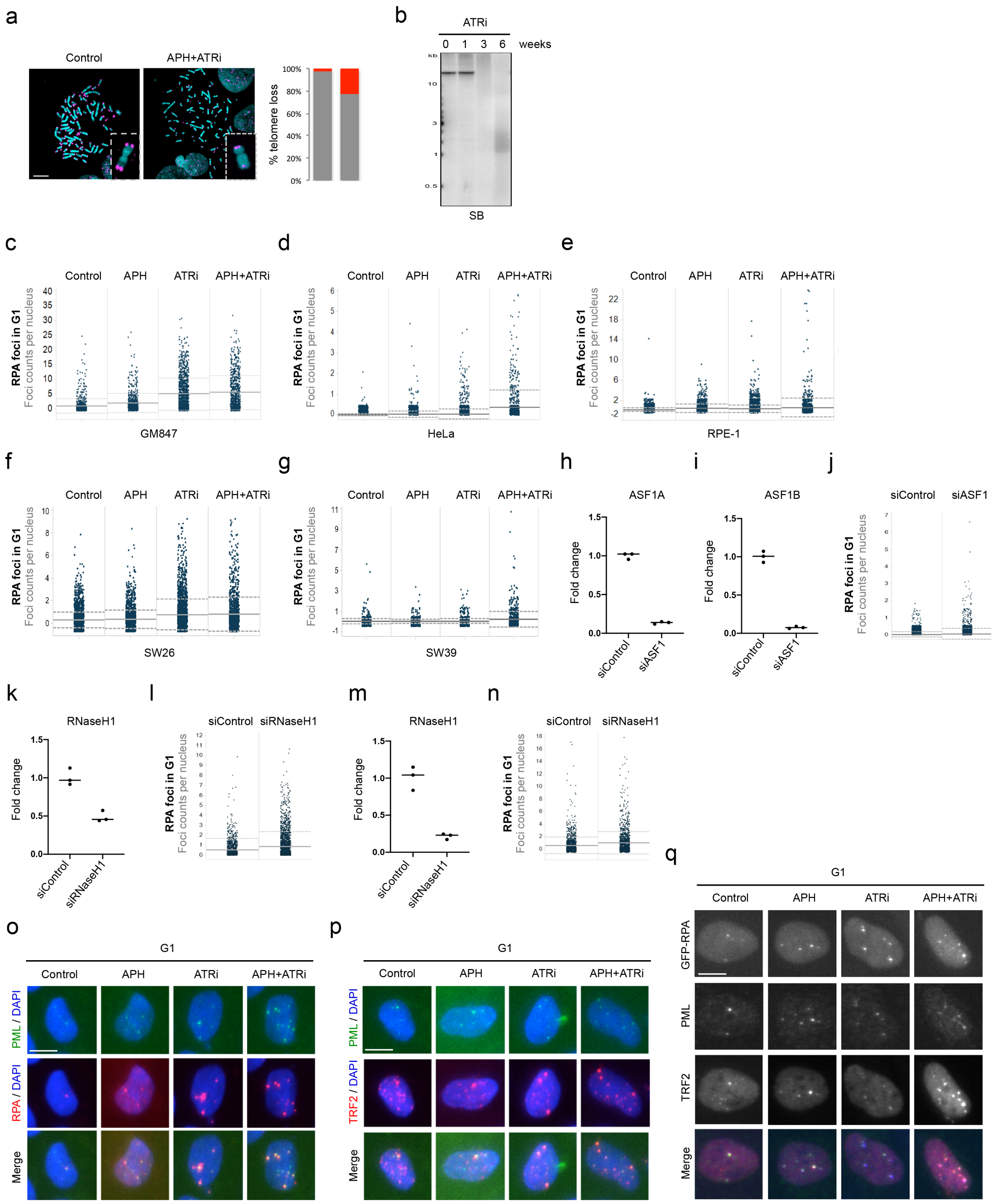

Supplementary Figure 8: ALT-positive cancer cells are particularly prone to inherit telomere lesions from the previous cell cycle. (a) Representative telomere FISH images from U-2 OS cells showing fragile telomeres on metaphase spreads upon APH+ATRi treatment for 24h. The percentage of metaphase chromosomes with telomere loss was quantified from ≥ 850 chromosomes per condition. **(b)** Southern blot analysis of telomeric DNA from U-2 OS cells treated continuously with a low concentration of ATRi (0.1μM) for several weeks as indicated. **(c-g)** Quantification of RPA foci in G1 in **(c)** ALT-positive GM847 cells from at least 500 G1 cells per condition; **(d)** ALT-negative HeLa cells from at least 1000 G1 cells per condition; **(e)** ALT-negative RPE-1 cells from at least 500 G1 cells per condition; **(f)** ALT-positive SW26 cells from at least 1000 G1 cells per condition; **(g)** ALT-negative SW39 cells from at least 500 G1 cells per condition. Horizontal lines indicate means ± SD of single cell data. **(h-i)** Knockdown controls by qRT-PCR for **(h)** ASF1A and **(i)** ASF1B depletion in HeLa cells. Data represent means ± s.d.; n=3 technical replicates. **(j)** Quantification of RPA foci in siControl and siASF1 HeLa cells (5 days of depletion). At least 1000 G1 cells per condition were analysed. Horizontal lines indicate means ± SD of single cell data. **(k)** Knockdown control by qRT-PCR for RNaseH1 depletion in U-2 OS cells. Data represent means ± s.d.; n=3 technical replicates. **(l)** Quantification of RPA foci in at least 1000 G1 cells per condition in siControl and siRNaseH1 U-2 OS cells. Horizontal lines indicate means ± SD of single cell data. **(m)** Knockdown control by qRT-PCR for RNaseH1 depletion in GM847 cells. Data represent means ± s.d.; n=3 technical replicates. **(n)** Quantification of RPA foci in G1 in siControl and siRNaseH1 GM847 cells. At least 1000 G1 cells per condition were analysed. Horizontal lines indicate means ± SD of single cell data. **(o-p)** U-2 OS cells were treated as indicated and stained for **(o)** RPA and PML and **(p)** TRF2 and PML. G1 cells were identified by QIBC. Representative images of G1 cells are shown. **(q)** U-2 OS GFP-RPA cells were treated as indicated and stained for TRF2 and PML. Representative images of G1 cells are shown. Scale bars: 10μm. A. U., arbitrary units. Source data are provided as a Source Data file.

Supplementary Figure 9

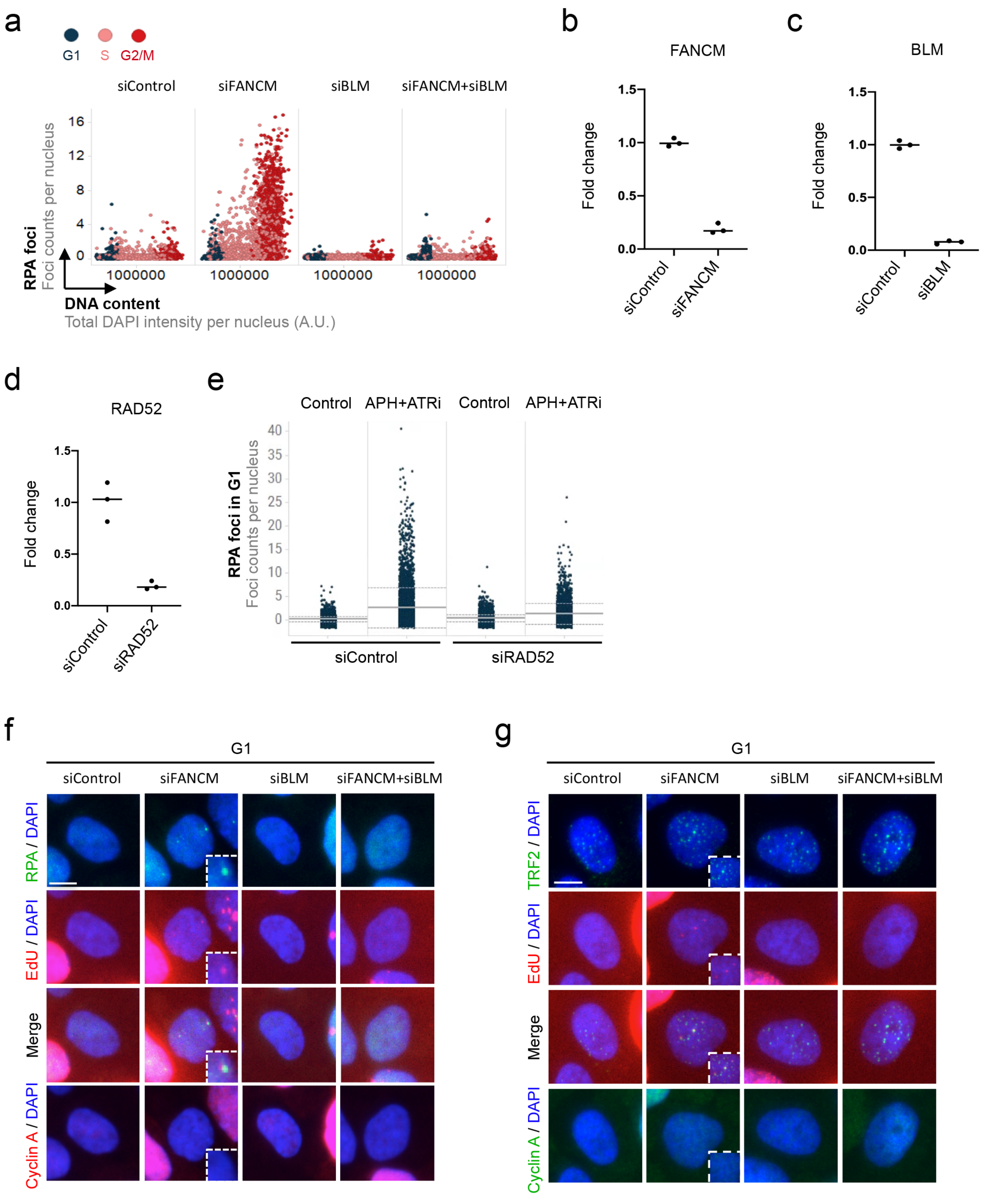

Supplementary Figure 9: RPA-marked lesions are primed for post-mitotic DNA synthesis (post-MiDAS). (a) To induce replication stress at telomeres, U-2 OS cells were depleted of FANCM for 48h. As telomere replication stress after FANCM loss depends on BLM, also BLM was depleted, either alone or together with FANCM as indicated. QIBC-derived cell cycle resolved profiles of RPA foci are shown. At least 1000 cells per condition were analysed. **(b)** Knockdown control by qRT-PCR for FANCM depletion in U-2 OS cells. Data represent means ± s.d.; n=3 technical replicates. **(c)** Knockdown control by qRT-PCR for BLM depletion in U-2 OS cells. Data represent means ± s.d.; n=3 technical replicates. **(d)** Knockdown control by qRT-PCR for RAD52 depletion in U-2 OS cells. Data represent means ± s.d.; n=3 technical replicates. **(e)** Quantification of RPA foci in G1 in siControl and siRAD52 U-2 OS cells treated as indicated. At least 1000 G1 cells per condition were analysed. Horizontal lines indicate means ± SD of single cell data. **(f)** U-2 OS cells were transfected and treated as in (a), G1 cells were identified by QIBC based on DNA content, Cyclin A and EdU, and images of G1 cells with EdU-positive RPA foci are shown. **(g)** U-2 OS cells were transfected and treated as in (a), G1 cells were identified by QIBC based on DNA content, Cyclin A and EdU, and images of G1 cells with EdU-positive TRF2 foci are shown. Scale bars: 10µm. A. U., arbitrary units. Source data are provided as a Source Data file.

Supplementary Figure 10

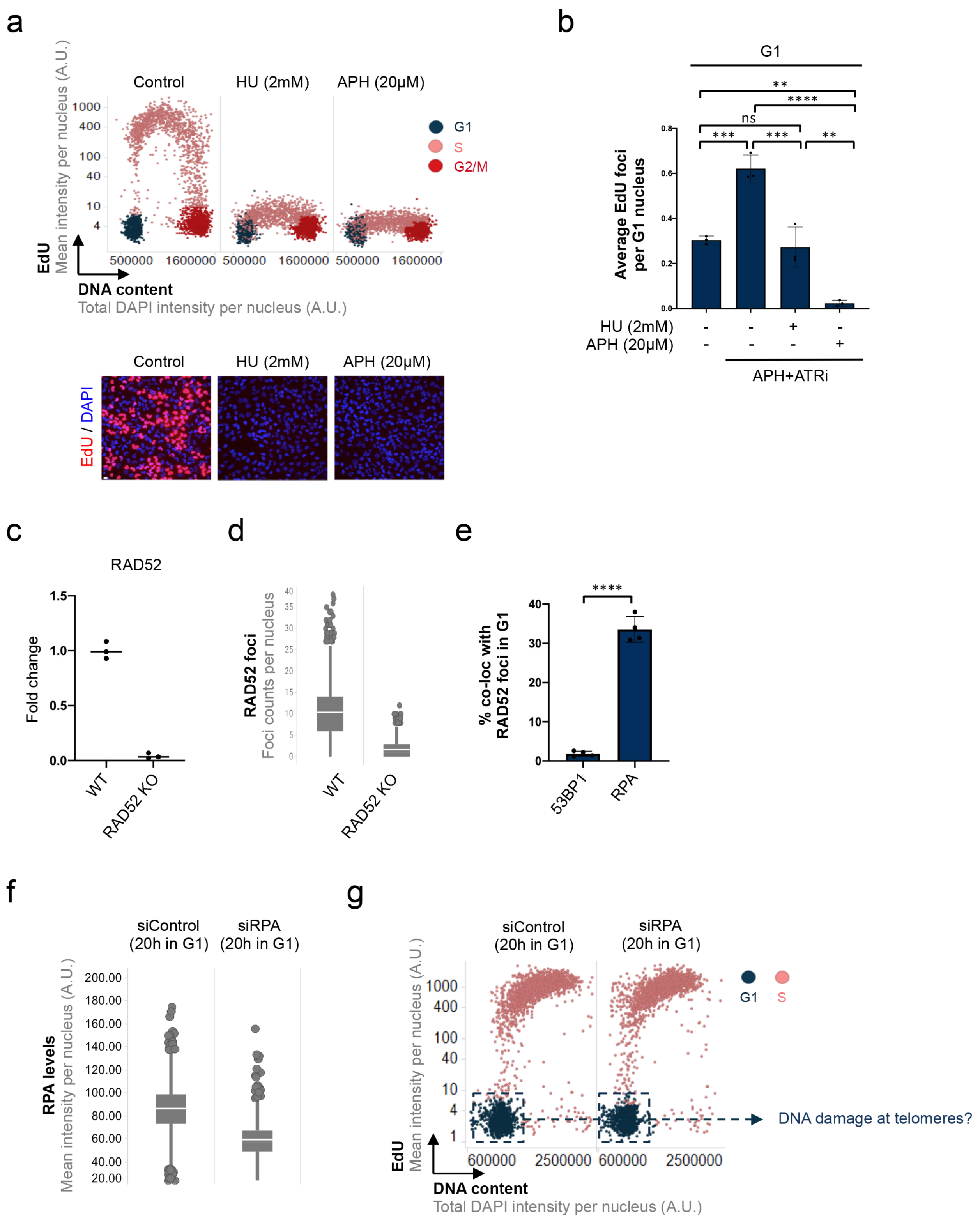

Supplementary Figure 10: RPA protects ALT telomeres during post-MiDAS from breakage. **(a)** EdU incorporation in U-2 OS cells after high doses of HU (2mM) or APH (20µM) for 2h, with EdU added for the last hour (20µM). QIBC-derived cell cycle resolved EdU levels are shown. At least 1000 cells per condition were analysed. Representative images of the EdU incorporation QIBC data are shown below. **(b)** Average EdU foci (100µM, 1h pulse) per G1 nucleus in cells treated with high doses of HU or APH as in (a) from n=3 independent samples with n_1=134, n_2=301, n_3=157 (Control), n_1=366, n_2=345, n_3=376 (APH+ATRi), n_1=336, n_2=363, n_3=350 (APH+ATRi+HU), n_1=362, n_2=328, n_3=293 (APH+ATRi+APH high dose) cells in G1 per sample. Individual values and means ± SD are shown. P-values were determined by one-way analysis of variance (ANOVA) with Tukey's test; ** p<0.01 (exact p-values are p=0.0011 and p=0.0024, respectively), *** p≤0.001 (exact p-values are p=0.0005 and p=0.0002, respectively), **** p≤0.0001, ns p≥0.05 (exact p-value is p=0.8929). **(c)** Knockout control by qRT-PCR for RAD52 in RAD52 KO U-2 OS cells. Data represent means ± s.d.; n=3 technical replicates. **(d)** RAD52 antibody specificity control by QIBC in RAD52 KO U-2 OS cells with n=1911 (WT), n=1922 (KO) cells per condition. Box plot with means is shown, boxes indicate the 25th and 75th centiles, whiskers indicate 5 and 95 per cent values. **(e)** Quantification of co-localization in U-2 OS cells between RAD52 foci and 53BP1 foci versus RAD52 foci and RPA foci in G1 cells from n=4 independent samples with n_1=233, n_2=202, n_3=360, n_4=347 for RAD52 and 53BP1 and n_1=455, n_2=412, n_3=566, n_4=477 for RAD52 and RPA. Individual values and means ± SD are shown. P-values were determined by two-tailed unpaired t-test; **** p≤0.0001. **(f)** U-2 OS cells were arrested in G2/M by nocodazole, a mitotic shake-off was performed and cells were released in fresh medium for 4h, allowing them to pass through mitosis. RPA or control siRNA depletion was then performed for 20h in presence of CDKi RO-3306 (to block remaining S/G2 cells from cell division) and EdU (to mark cells entering S-phase and to be able to discriminate them from G1 cells). The numbers of cells analyzed are n=1547 (siControl) and n=1385 (siRPA). Box plot with mean RPA intensities after 20h of depletion are shown, boxes indicate the 25th and 75th centiles, whiskers indicate 5 and 95 per cent values. **(g)** As in (f), cells were arrested in G2/M and released in fresh medium for 4h. RPA depletion was then performed for 20h in presence of CDKi RO-3306 (to block remaining S/G2 cells from cell division) and EdU (to mark cells entering S-phase and to be able to discriminate them from G1 cells). QIBC-derived EdU profiles are shown. At least 1000 cells per condition were analysed. Scale bars: 10µm. A. U., arbitrary units. Source data are provided as a Source Data file.

Supplementary Table 1: List of cloning primers

ID	Name	Sequence
1	pUC18 F	5'-AGGATCCCCGGGTACCGAGCT-3'
2	pUC18 R	5'-CTAGAGTCGACCTGCAGG-3'
3	RPA70 800 F	5'-TGCATGCCTGCAGGTCGACTCTAGGGTCTCGCCCGTTCTTCTGCGACTAC-3'
4	RPA70 800 R	5'-CGAGCTCGGTACCCGGGGATCCTGGTCTCGGCAAGTCAATGCTTATCAGC-3'
5	pUC18 RPA70 800 F	5'-GAGGAGCAGTGCCAATCGGGCAG-3'
6	pUC18 RPA70 800 R	5'-CATCAATGCACTTCTCCTGATGCTC-3'
7	mScarlet-P2A-Blast F	5'-GGTCATGAGCATCAGGAGAAGTGCATTGATGGGAGCTGGTGCAGGCGC-3'
8	mScarlet-P2A-Blast R	5'-CAAACTTCTGCCCGATTGGCACTGCTCCTCTTAGCCCTCCCACACATAAC-3'
9	RPA70 gRNA F	5'-CACCGTTTGCAAACTTCTGCCCGATTGG-3'
10	RPA70 gRNA R	5'-AAACCCAATCGGGCAGAAGTTTGCAAAC-3'
11	RPA70-mScarlet F	5'-GACGAGTCTCGAATTAAGGCC-3'
12	RPA70-mScarlet R	5'-CCACTGGAGAAGCAAAAACC-3'

Supplementary Table 2: List of qPCR primers

Name	Sequence
hASF1A Forward	5'-AGTGCATCGAGGACCTGTCTGA-3'
hASF1A Reverse	5'-TGCGGGAACAGGACCCACTAAA-3'
hASF1B Forward	5'-TGGACAGGAGTTCATCCGAGTG-3'
hASF1B Reverse	5'-GTCCCAGTTGATATGGAAGCGG-3'
hBLM Forward	5'-CCTCCCAAAGGTCTAAGAGGA-3'
hBLM Reverse	5'-GATATCTTTCTACATGTGGCAGACC-3'
hFANCM Forward	5'-GCCGTAAACGTCAAGGCAGGAT-3'
hFANCM Reverse	5'-CCATCAGGAACCATTCGTGGAC-3'
hRAD52 Forward	5'- GCCCAGAATACATAAGTAGCCGC-3'
hRAD52 Reverse	5'-CCACATTCTGCTGCGTGATGGA3'
hRNASH1 Forward	5'-CCTCCAGTTAGCAGAGACACGT-3'
hRNASH1 Reverse	5'-CCAGTAAACGCCGATTCCTGC-3'
hRPS12 Forward	5'-GGAGGCTTGGGTGCGTTC-3'
hRPS12 Reverse	5'-GGTGGCAGTTTTGTTCCG-3'
