# Supplementary material for: RPA shields inherited DNA lesions for post-mitotic DNA synthesis
Source: Nat Commun. 2021 Jun 22;12:3827. doi: 10.1038/s41467-021-23806-5 (PMC8219667; doi:10.1038/s41467-021-23806-5)
Supplement: Supplementary file 3 — Description of Additional Supplementary Files [file 41467_2021_23806_MOESM3_ESM.pdf]

Description of Additional Supplementary Files

File Name: Supplementary Movie 1
Description: **53BP1 and RPA mark distinct heritable DNA lesions.**
Corresponds to Supplementary Figure 3c. Untreated asynchronously growing U-2 OS 53BP1-GFP / RPA70-mScarlet cells were followed through mitosis and into G1 by time-lapse microscopy at 30 minute intervals. Scale bars: 10µm.

File Name: Supplementary Movie 2
Description: **RPA-marked heritable DNA lesions can be followed through a complete cell cycle.**
Corresponds to Supplementary Figure 3d. Untreated asynchronously growing U-2 OS RPA-GFP cells were followed through the cell cycle by time-lapse microscopy at 30 minute intervals. Scale bars: 10µm.
